# High-throughput micro-phenotyping measurements applied to assess stalk lodging in maize (*Zea mays* L.)

**DOI:** 10.1186/s40659-018-0190-7

**Published:** 2018-10-27

**Authors:** Ying Zhang, Jianjun Du, Jinglu Wang, Liming Ma, Xianju Lu, Xiaodi Pan, Xinyu Guo, Chunjiang Zhao

**Affiliations:** Beijing Key Lab of Digital Plant, Beijing Research Center for Information Technology in Agriculture, Beijing Academy of Agriculture and Forestry Sciences, Shuguang Huayuan Middle Road, Haidian District, No. 11, Beijing, 100097 People’s Republic of China

**Keywords:** Maize stalk, Stalk lodging, Anatomical phenotypes, Vascular bundle, VesselParser 2.0

## Abstract

**Background:**

The biomechanical properties of maize stalks largely determine their lodging resistance, which affects crop yield per unit area. However, the quantitative and qualitative relationship between micro-phenotypes and the biomechanics of maize stalks is still under examined. In particular, the roles of the number, geometry, and distribution of vascular bundles of stalks in maize lodging resistance remain unclear. Research on these biomechanical properties will benefit from high-resolution micro-phenotypic image acquisition capabilities, which have been improved by modern X-ray imaging devices such as micro-CT and the development of micro-phenotyping analysis software. Hence, high-throughput image analysis and accurate quantification of anatomical phenotypes of stalks are necessary.

**Results:**

We have updated VesselParser version 1.0 to version 2.0 and have improved its performance, accuracy, and computation strategies. Anatomical characteristics of the second and third stalk internodes of the cultivars ‘Jingke968’ and ‘Jingdan38’ were analyzed using VesselParser 2.0. The relationships between lodging resistance and anatomical phenotypes of stalks between the two different maize varieties were investigated. The total area of vascular bundles in the peripheral layer, auxiliary axis diameter, and total area of vascular bundles were revealed to have the highest correlation with mechanical properties, and anatomical phenotypes of maize stalk were better predictors of mechanical properties than macro features observed optically from direct measurement, such as diameter and perimeter.

**Conclusions:**

This study demonstrates the utility of VesselParser 2.0 in assessing stalk mechanical properties. The combination of anatomical phenotypes and mechanical behavior research provides unique insights into the problem of stalk lodging, showing that micro phenotypes of vascular bundles are good predictors of maize stalk mechanical properties that may be important indices for the evaluation and identification of the biomechanical properties to improve lodging resistance of future maize varieties.

## Background

Plant phenotyping is defined as the application of methodologies and protocols to measure specific traits related to plant structure and function, including traits at the cellular, organ, whole plant, and even canopy levels [1, 2]. Micro-phenotyping focusing on the cellular and tissue levels plays a vital role in estimating genotype–phenotype relationships and, consequently, in improving the efficiency of breeding. Operating at a lower throughput, micro-level phenotyping requires more complex processes, such as notoriously time-consuming and laborious sample preparation and high-resolution microscopic image acquisition. Moreover, quantitative analysis of anatomical characteristics based on micrographs tends to lack automated and adaptive image analysis pipelines. Previous analyses have heavily relied on human researchers at each step, including image segmentation, feature extraction, and classification [3]. Obviously, for regions of interest (ROI), manual segmentation is not only labor intensive, but is restricted by human subjectivity and inconsistency that may severely limit the throughput and precision of micro-phenotypic trait analyses. Hence, high-throughput and accurate analysis methods are strongly needed [4, 5].

Microscope imaging techniques and the introduction of machine learning in cell biology have boosted plant phenotyping at the cellular level [5]. The machine learning methods used in microscopic image analysis mainly include neural networks [6], adaptive lifting [7], random forests [8], and support vector machines [9]. Because of the large differences in phenotypic characteristics of specific cells, organs and crops, targeted development or secondary development is required to meet current research needs, which has led to the continuous emergence of a large number of algorithms and tools in recent years. The related software packages and toolkits that have been reported include Wndchrm [10], EB Image [11], CellProfiler [12], PhenoRipper [13], and Fiji [14]. These software packages were used to conduct automated cell segmentation and measurement or as an aid in cell trait measurement of confocal, laser dissection micrographs of *Arabidopsis* *thaliana*, wheat (*Triticum* spp.), and maize (Zea *mays*) roots [15–18]. Anatomical phenotyping of stalks is much more complicated than that in root tissue, which increases the difficulty in developing accurate microscopic phenotyping analysis pipelines for stalks. Previous research has reported methods for lignification quantification of maize tissues and analyzing numbers of vascular bundles mainly by automated color image analysis of stained maize stalk cross sections [19–21]. In 2016, Du et al. first explored a specific image analysis method for quantifying total vascular bundle phenotypic traits based on micro-CT cross-sectional images, and they implemented this methodology in the VesselParser 1.0 software package [22]. VesselParser 1.0 enabled the automatic analysis of phenotypic traits of all vascular bundles within an entire cross-section of maize stalk. Based on this encouraging result, we have enhanced and improved the functionality of this software. Based on VesselParser 1.0 and our routine test applications, we found that more elaborate extractions of geometric features, statistical post-analyses, and batch-processing were needed.

Stalk lodging in maize is a major agronomic problem that has far-reaching economic ramifications. Many studies have found that stalk mechanical properties were positively correlated with stalk lodging resistance in the field [23–27]. A better understanding of mechanical properties in maize will make stalks stronger and ultimately reduce yield and grain quality losses [28]. This biomechanical property has been shown to be obviously related to weather, environmental conditions, chemical composition, and stalk morphology. Variation in mechanical support performance of stalks has been reported among varieties or different mutant lines [29–32], and considerable research has focused on the relationship between biomechanical properties and physical soil characteristics, plant-growth parameters, and meteorological factors [33]. However, the culminating event in stalk lodging is structural failure (i.e., breakage) of the stalk, which should be more immediately relevant to the anatomical structure and composition of stalk, including their geometry, quantity, and distribution of lignin and vascular bundles. Considerable progress has been made in understanding the effects of stalk morphology (i.e., diameter), vascular bundle system development and anatomical composition, and lignin traits on biomechanical properties [19, 34–36].

Vascular bundles are the key component of maize stalks. Previous studies have shown that the micro-phenotypic traits of vascular bundles, e.g., their distribution and proportion of rind to total stalk tissue in gramineous crops, affects their mechanical properties [37–39]. However, the sophisticated quantitative analysis of stalk anatomical phenotypes is still the limiting factor in understanding stalk mechanical properties because many vascular bundle anatomical properties, such as vascular bundles area, vascular bundle distribution density, etc., are difficult to manually measure. Hence, a practical method suitable for high-throughput and highly accurate quantification of anatomical features of maize stalks and the geometric distribution of vascular bundles is greatly needed.

**Fig. 1:**
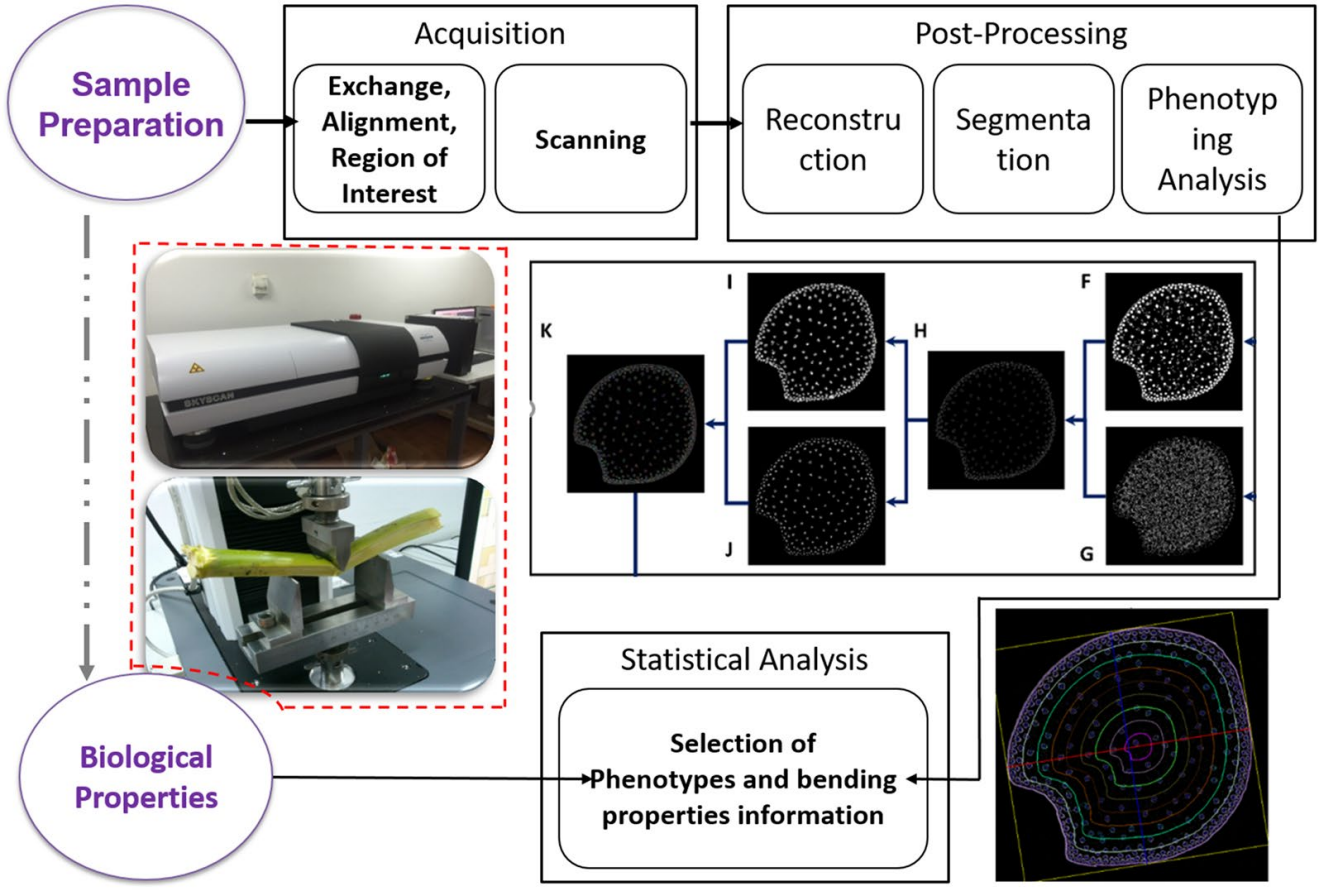
Schematic diagram of sample micro-CT scanning, three-point bending test, and the subsequent statistical analysis

This study aimed to identify the associations between anatomical phenotypes and the potential for stalk lodging. The multidisciplinary approach implemented in this study represents collaborative research that integrates image processing, biological information, and plant science. The specific results of this study include the following: (i) an upgraded VesselParser version 2.0 image processing pipeline; (ii) high-throughput and highly accurate quantification of anatomical phenotypes of stalk base internodes for different varieties; (iii) an evaluation of the stalk mechanical properties of different varieties, (iv) an investigation of the relationships between stalk anatomical phenotypes and plant lodging properties based on the phenotyping dataset. Ultimately, the optimal model contained macro- and micro-phenotypic parameters that were closely related to maize stalk mechanical properties (Fig. 1).

## Methods

### Plant material and growth conditions

Seeds for the maize cultivars ‘Jingdan38’ (abbreviated as JD38) and ‘Jingke968’ (abbreviated as JK968) were planted at the Beijing Academy of Agriculture and Foresting Sciences in Beijing, China. Rows were 60 cm apart with a density of 60,000 plants/ha. Sowing took place on May 11, 2016. The soil was tilled to a depth of 15 cm before sowing, and the soil texture was loamy sand with a field capacity of 32% in the plow layer. Other chemical properties of the plow layer were as follows: 27.2 g/kg organic matter, 1.34 g/kg total N, 37.6 mg/kg available phosphorus, 91 mg/kg ammonium acetate extractable potassium, and pH 7.6.

**Fig. 2:**
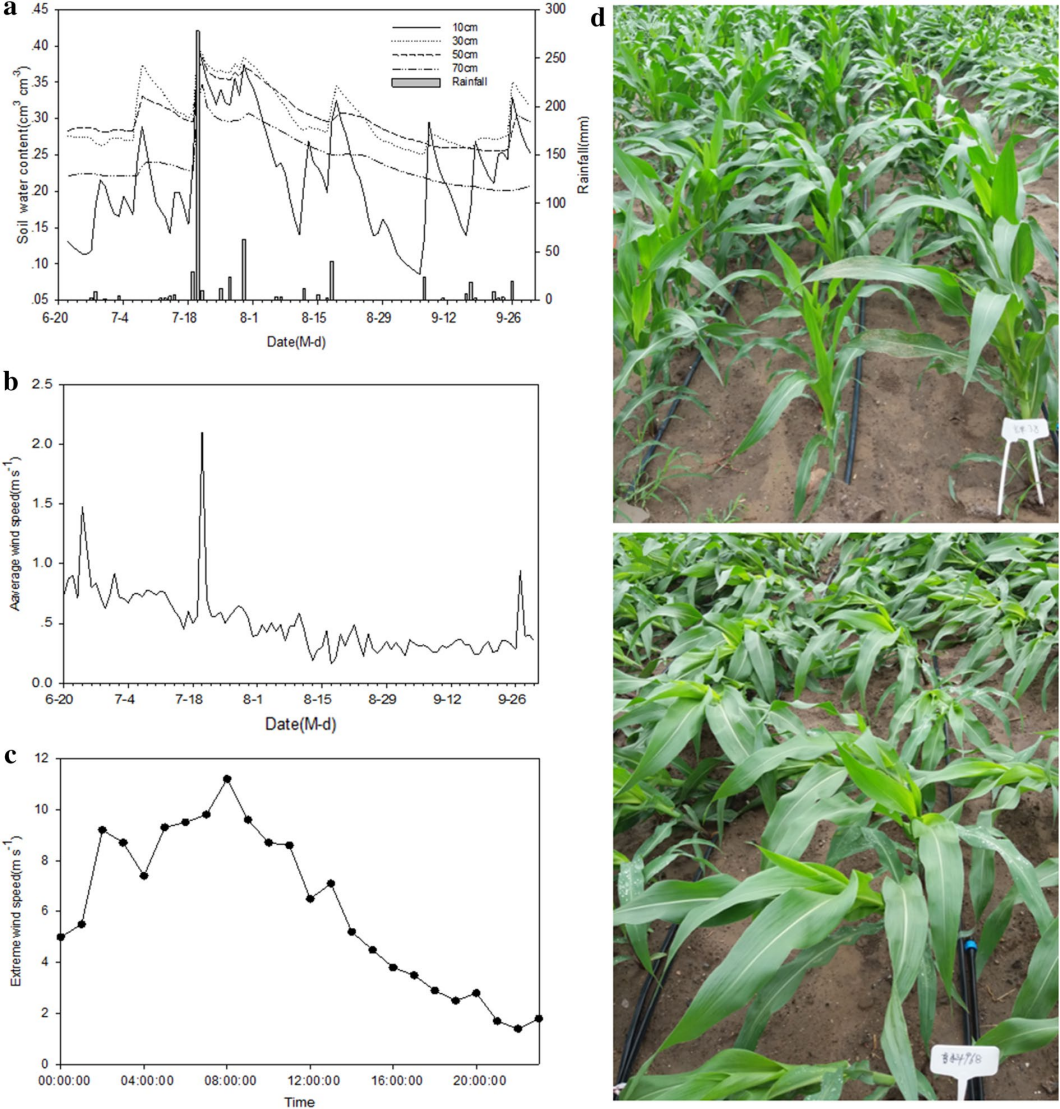
Meteorological information and plant lodging observed in the study site. **a**–**c** Meteorological data from the meteorological station installed at the study site, **d** images of the two maize varieties after a strong wind and rain storm

Meteorological data were obtained from the meteorological station installed at the study site. The total precipitation was 287.8 mm from July 20–21, and the maximum wind speed was 11.2 m·s^−1^. The soil water reached a saturated water content of 44% and appeared waterlogging on the soil surface on July 21. Strong wind and rain storm conditions were associated with stalk lodging. Notably, there were significant differences in lodging between the two varieties. Almost all JK968 plants were blown down, but there were negligible effects on JD38 plants, which remained standing upright as shown in Fig. 2. Twenty plants of each variety were selected randomly for three-point bending tests and micro-CT scanning.

### Evaluation of stalk bending properties

Twenty plants of each variety were randomly excavated for analysis after strong winds and heavy rain on July 22. At this point, the maize was at the 13-leaf stage. The bending properties of the second and third stalk internodes of two different field-grown maize varieties were quantified. Stalks were placed between moist germination papers and refrigerated at 4 °C for 24 h to preserve them until measurement in order to minimize degradation. Three-point bending tests were carried out at Beijing Key Lab of Digital Plant, Beijing Academy of Agricultural and Forestry Sciences. An Instron 3340 (Norwood, Massachusetts, USA) test frame equipped with a 500 N load cell (Instron 2519-104 Series, Norwood, Massachusetts, USA) and a standard loading anvil and supports were used to conduct all tests. Samples were placed between two supports (set apart 3 cm) and the loading anvil was displaced at a rate of 20 mm/min. Load–displacement data were recorded every 100 ms just until failure was detected. Failure was defined as the first instance of a decrease in load-bearing capacity (i.e., stalk breakage) [40].

### Stalk micro-CT image acquisition

After the three-point bending tests, the undamaged parts of the second and third stalk internodes for JD38 and JK968 plants were collected and cut into 1.0–1.5 cm segments. Sample preparation was performed according to the method described in 2016 [22]. Dried samples were scanned using Skyscan 1172 X-ray computed tomography system (Bruker Corporation, Billerica, MA, USA) employing a 40 kV/250 μA tungsten X-ray source and a 1.3 megapixel cooled CCD camera. The object to source was 259.850 mm, the camera to source was 345.591 mm for all individuals. The obtained raw CT data in the 4 K scan module were converted into a series of CT slice images in 8-bit tagged image bitmap (BMP) format using Skyscan NRecon software (Bruker Corporation). Each variety contained 20 replicates.

### Image analysis strategies

VesselParser software was developed for high-throughput detection of the anatomical phenotypes of maize stalks based on high-resolution micro-CT images. We updated the software from version 1.0–2.0 to improve the performance, accuracy, and computation strategies, including improvement of image segmentation, performance, and analysis precision.

#### Image segmentation

The overall goal was to enable a researcher to extract useful quantitative information from large numbers of microscopic images of basal stalk internodes, enabling the captured information to then be modified. Biological tissues of interest, such as epidermis, vascular bundles, slice layers, etc., were represented by different geometrical objects, and then these objects were combined into a data structure for maize stalks. Closed outer contours were used to represent complex shapes of tissues, and hierarchical inner closed contours were utilized to represent complex tissues with embedded objects. In brief, a vascular bundle could be represented by an individual contour or a combination of more than two contours, dependent upon the segmentation and classification results of vascular bundles. The data representation of vascular bundles were designed as a common class, in which those properties consisted of the outer contour, inner contours, owner layer, uniform ID, name string, size type, and spline node number, among others. Moreover, a set of simple threshold-based segmentation and morphological operations can determine the outermost boundary and average thickness of epidermis (the outside layer of maize stalks). From the inside boundary of epidermis, series of virtual equidistance layers with predefined numbers can also be generated. These new virtual layers were used to decompose the distribution of vascular bundles into meaningful statistical regions, and the center position of the last layer was in accord with the central maize stalk layer.

#### Performance improvement

VesselParser software was implemented using Visual C++ and an open source computer vision library (OpenCV, https://opencv.org/). The whole image processing workflow had the following advantages compared with the previous version. First, high-resolution CT images of large-scale samples were loaded from a user-specified file directory, and these image files were fed to the image analysis pipeline in batches. Since each image represented an individual task, the parallel computation scheme could simultaneously handle with several tasks according to the computer configuration. Second, each individual task was executed automatically according to uniform algorithm parameters to extract phenotypic traits of interest from the corresponding high-resolution CT image. Third, each computation result was serialized as a local binary file format (named as.VBF file) which stored the integrated information of the slice and vascular bundles. After the phenotyping computation, these computation results could be reloaded and collected for statistical analysis. This workflow guarantees the consistency and computation efficiency of the image processing pipeline. The batch processing, parallel computation and data serialization significantly improve the software performance, and achieve data processing capacity of 10 slice images per minute, which make it possible to automatically detect the micro phenotypes from large-scale slice samples of different varieties.

#### Precision improvement

**Fig. 3:**
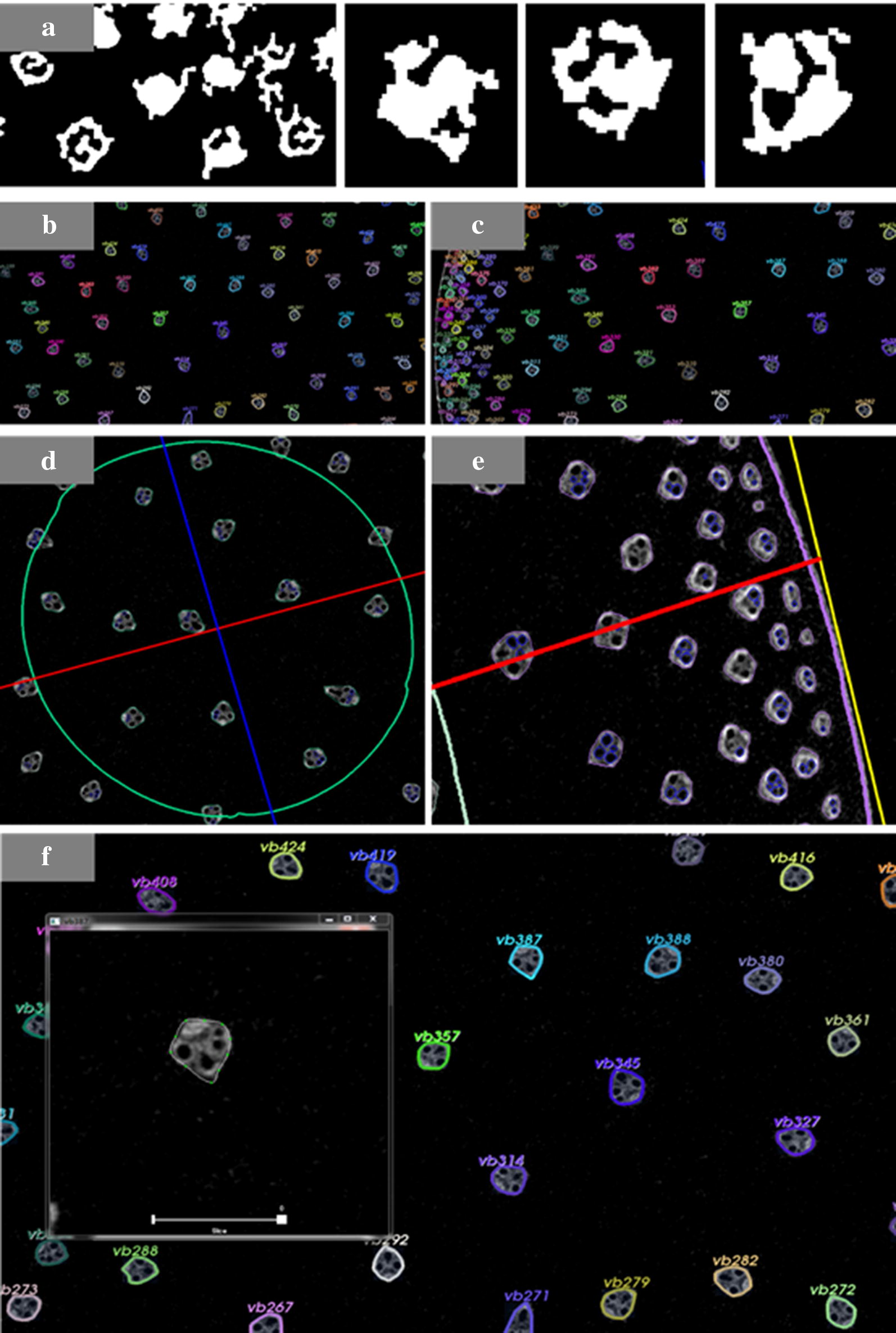
Phenotypic traits analysis of vascular bundles in maize stalks using by Vessel Parser 2.0. **a** Vascular bundles segmented by the fixed or adaptive threshold methods. **b** Vascular bundles located in the center region. **c** Vascular bundles located at the boundary region, **d** inner layer, **e** and outer layer. **f** The spline modification for individual vascular bundles

The rind of base internodes is rich in collenchyma, and the peripheral vascular bundles are usually visibly distinct. Higher distribution densities and differing morphological structures are great challenges for high-throughput, automated, and accurate phenotypic trait detection by VesselParser 1.0. In the segmentation of vascular bundles, ambiguity was a common issue, regardless of the fixed or adaptive threshold (Fig. 3a). Owing to the variation in vascular bundles and imaging qualities, the contours resulting from image segmentation did not truly represent the content and shape characteristics of vascular bundles. Therefore, a new three-step scheme was developed. First, each individual segmented object must exceed the validation-based shape and area ratio threshold, but the filtering parameters are also related to its distance from the shape centroid. Second, the convex hull of each segmented object was generated according to its outer contour. After validation verification, this convex hull, consisting of fewer points, could be taken as the outer contour of this segmented object. Third, the outer contour was fitted to a closed SPL spline with user-predefined control points. Obviously, this spline would assist users to subtly edit and adjust the shape of the contour (Fig. 3f). The outer contour and its spline representation need to be synchronously updated, and the phenotyping analysis can then be executed based on the modified shape. Primary phenotypic traits are listed in Table 1. We divided the cross-section of the maize stalk into four individual layers of equal area and sampled the peripheral layer, central second layer, central third layer, and central fourth layer from the outside to inside. A layer map with concentric contours was utilized to interpret the spatial distribution of vascular bundles at the stalk internodes.

**Table 1:** Twenty-one anatomical phenotypes measured or derived using VesselParser 2.0 on micro-CT scanned cross-section image

Traits by tissue region	Abbreviation
Stalk cross-section	
Principal axis diameter (mm)	PAD
Auxiliary axis diameter (mm)	AAD
Circumradius (mm)	CCR
Section area (mm^2^)	SA
Vascular bundles	
Vascular bundle number	VB
Vascular bundle number of peripheral layer	PVB
Vascular bundle number of central 2nd layer	CVB-2
Vascular bundle number of central 3rd layer	CVB-3
Vascular bundle number of central 4th layer	CVB-4
Vascular bundle total area in peripheral layer (mm^2^)	PA
Vascular bundle total area in central 2nd layer (mm^2^)	CA-2
Vascular bundle total area in central 3rd layer (mm^2^)	CA-3
Vascular bundle total area in central 4th layer (mm^2^)	CA-4
Vascular bundle area ratio of peripheral layer	PAR
Vascular bundle area ratio of central 2nd layer	CAR-2
Vascular bundle area ratio of central 3rd layer	CAR-3
Vascular bundle area ratio of central 4th layer	CAR-4
Vascular bundle average area (mm^2^)	MA
Vascular bundle total area (mm^2^)	TA
Ratio of vascular bundle total area and section area	Rsection
Aspect ratio	AR

### Data analysis

The R statistical computing environment (R Development Core Team 2014) was used for data analysis. First, principal components analysis (PCA) was performed on the original data of 21 phenotypic traits in order to describe correlation patterns among phenotypes, which could help illustrate the relationships among all traits simultaneously and summarize them in one graph. Then, Pearson’s correlation analysis was used to test for relationships between stalk mechanical properties and stalk anatomical phenotypes. Besides, we performed correlation test to validate the significance of the correlations of variables. Subsequently, traits which significantly (*P* value < 0.05) correlated to mechanical properties were used as independent variables in a multiple linear regression to identify their contribution to bending strength. Stepwise multiple linear regression was used to identify the traits contributing to stalk bending strength. The model with the lowest Akaike information criterion (AIC) value was selected as the optimal mode for evaluating maize stalk bending strength. In addition, quality control of stalk samples was implemented with model diagnosis.

## Results

### Three-point bending tests for the two varieties

**Fig. 4:**
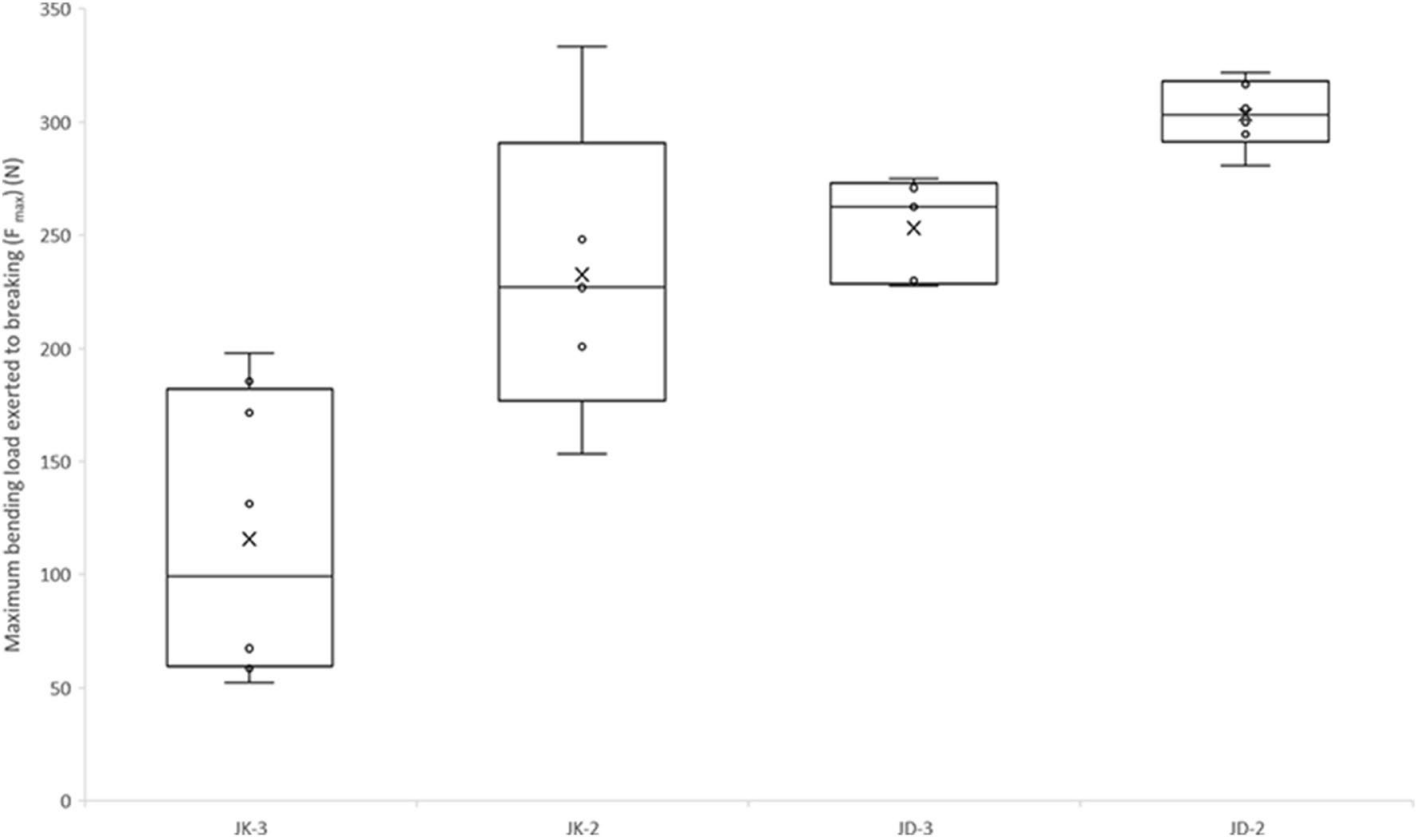
The maximum bending load exerted to breaking (Fmax) for different stalk internodes for the two different varieties: JK-3, boxplot depicting the Fmax of the third stalk internode for ‘Jingke968’; JK-2, boxplot depicting the Fmax of the second stalk internode for ‘Jingke968’; JD-3, boxplot depicting the Fmax of the third stalk internode for ‘Jingdan38’; JD-2, boxplot depicting the Fmax of the second stalk internode for ‘Jingdan38’

Violent weather caused plant lodging, with obvious differences between the two varieties. Almost all JK968 plants were blown down by the strong wind and rain storm, but the incident had very little impact on JD38 plants, most of which remained upright as shown in Fig. 2d. The lodging resistance differences between the two varieties were unexpected. In order to further assess the biomechanical properties of stalks, three-point bending tests for the second and third stalk internodes of the two different varieties were conducted. Fmax (the bending load at which the stalk breaks) of the tested samples ranged between 115 N and 300 N, exhibiting twofold variation in bending strength. The bending performance of the second internode samples was significantly higher than that of the third internodes, which might be related to the maturity of corn stalk (i.e., the degree of development of vascular bundles). The bending performances of the stalks significantly differed between the two cultivars. The maximum load exerted until the breaking point for JD38 stalks was significantly higher than that for JK968. The average bending load at the breaking point of the second internode for JD38 was 303 N, but the average bending load at the breaking point of the second internode for JK968 was only about 232 N (Fig. 4).

### Micro-phenotypic traits of stalks for the two varieties

**Fig. 5:**
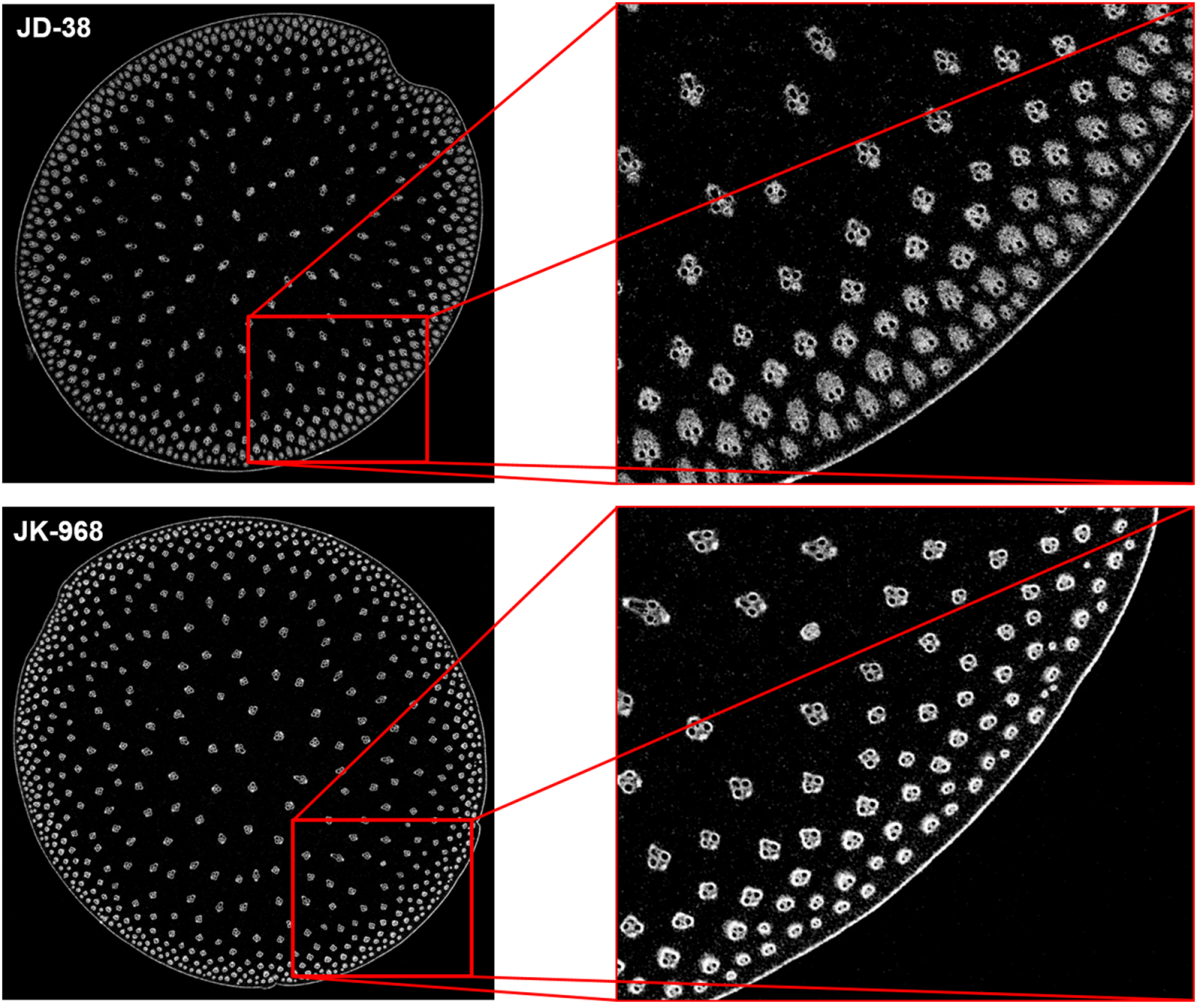
The micrographic characteristics of the cross-sectional stalk internodes for the two different varieties as measured from micro-CT scanning

The high-resolution micrographic images of JK968 and JD38 internodes were obtained via micro-CT scanning. In the stalk transactions, vascular bundles were clearly distributed more intensively in the rind but dispersedly in the center. Anatomical characteristics of stalk internodes differed substantially between JK968 and JD38 plants, especially in the rind region (the outmost layer) where vascular bundles were smaller and denser. Visually, the distribution density of vascular bundles in the rind region of JD38 plants was much denser than that of JK968 plants (Fig. 5). Larger and much more numerous vascular bundles were distributed in rind region of JD38 plants, while the area comprised of vascular bundles in JK968 plants was smaller with fewer bundles. Unlike the rind regions, the two varieties did not differ in the morphological characteristics of the vascular bundles of pith sections.

**Fig. 6:**
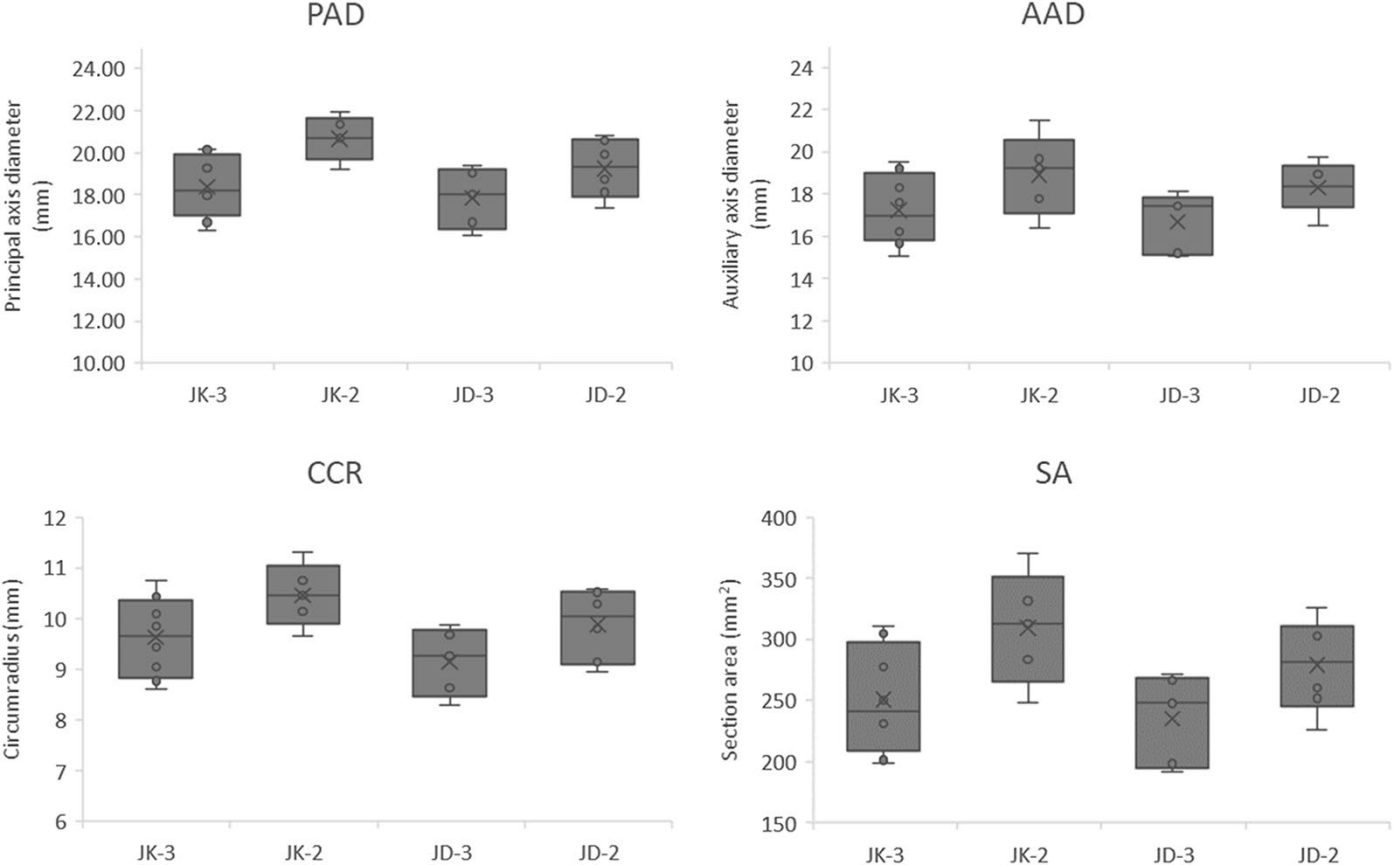
Boxplots for 4 extracted traits (PAD, AAD, CCR, and SA) illustrating the anatomical phenotypes of stalk cross-sections for two varieties

**Fig. 7:**
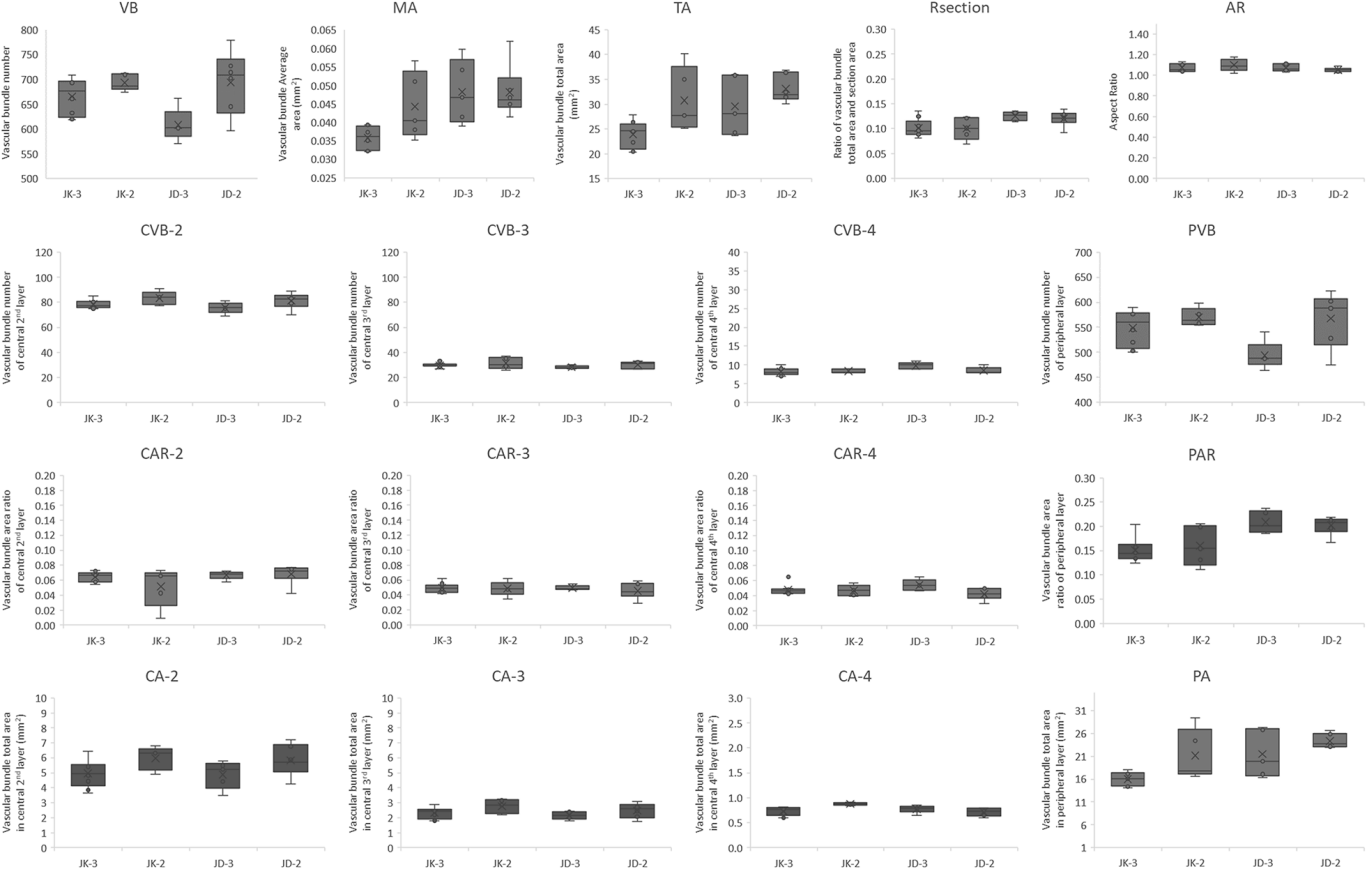
Boxplots for 17 extracted traits (VB, MA, TA, Rsection, AR, CVB-2, CVB-3, CVB-4, PVB, CAR-2, CAR-3, CAR-4, PAR, CA-2, CA-3, CA-4, and PA) illustrating the anatomical phenotypes of vascular bundles of stalk cross-sections for two varieties

In the study by Du et al., the fifth and above internodes sections were assayed using VesselPasser 1.0 software, finding that these materials significantly differed from the base internodes in structural characteristics such as vascular bundle number, shape, and distribution. The morphology of the peripheral vascular bundles in the fifth and above internodes is very regular. In contrast, the rind of the base internodes is rich in collenchyma, and the peripheral vascular bundles are usually visibly distinct. Higher distribution density and differing morphological structures present great challenges for high-throughput, automated, and accurate phenotypic trait detection with VesselPasser 1.0. In order to systematically and accurately obtain morphological and anatomical characteristics of different stalks, VesselParser 1.0 was improved to 2.0. VesselPasser 2.0 can be used to measure a total of 21 accurate anatomical parameters at one time: principal axis diameter (PAD), auxiliary axis diameter (AAD), circumradius (CCR), section area (SA), vascular bundle number (VB), vascular bundle number of the peripheral layer (PVB), vascular bundle number of the central second layer (CVB-2), vascular bundle number of the central 3rd layer (CVB-3), vascular bundle number of the central fourth layer (CVB-4), vascular bundle total area in the peripheral layer (PA), vascular bundle total area in the central second layer (CA-2), vascular bundle total area in the central third layer (CA-3), vascular bundle total area in the central fourth layer (CA-4), vascular bundle area ratio of the peripheral layer (PAR), vascular bundle area ratio of the central second layer (CAR-2), vascular bundle area ratio of the central third layer (CAR-3), vascular bundle area ratio of the central fourth layer (CAR-4), vascular bundle average area (MA), vascular bundle total area (TA), ratio of vascular bundle total area, section area (Rsection), and aspect ratio (AR; Figs. 6, 7). The performance improvement of VesselPasser 2.0 over its predecessor is clearest in four aspects: (1) more comprehensive phenotypic indicators relative to mechanical properties were available, with the number of phenotypic parameters of stalks increased from 14 to 21; (2) more accurate shape description and minimized shape modification can be conducted by man–machine interaction; (3) the potential prevention of smaller vascular bundles located in the boundary region being mistakenly deleted using the minimum area control, thereby increasing the accuracy of basal stalk internode estimates; (4) more efficient image analysis, such as batch processing and post-processing.

### Relationship between stalk mechanical properties and anatomical phenotypes of maize

**Fig. 8:**
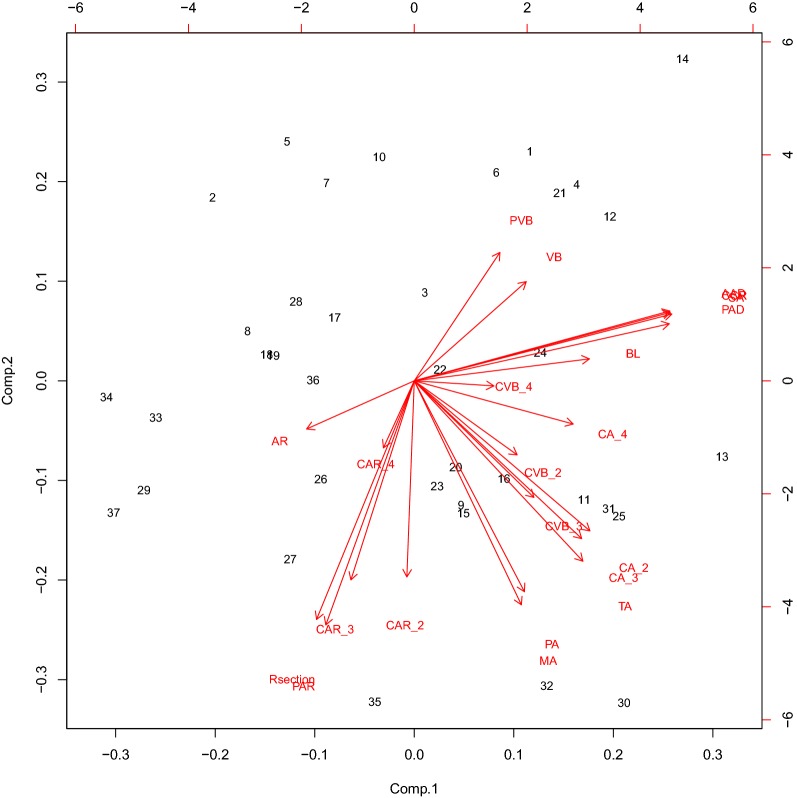
Principal component analysis (PCA) for 21 anatomical phenotypes among the two different varieties

In this study, 21 phenotypic parameters estimated from 37 samples (data from the remaining 3 samples were of poor quality and thus deleted) were used to compose a 37 × 21 matrix, and a principal component analysis was performed on this data using R software. The first six principal components accounted for 89.73% of the total variability, retaining the original variable information. Therefore, the first six components can be reliably considered the main components for distinguishing the phenotypic parameters. PCA showed that the first axis explained 30.96% of the variation among the 21 anatomical phenotypes and was mostly associated with PAD, SA, CCR, and AAD. The second axis explained 26.52% of the variation and was associated with PAR, CAR-3, Rsection, and CAR-4 (Fig. 8). Pearson’s correlation analysis was used to test for relationships between stalk mechanical properties and stalk anatomical phenotypes. Besides, we performed correlation test to validate the significance of the correlations of variables. The variables most associated with mechanical properties were PA, CAR-3, TA, PAL, AAL, CCR, SA, and AR (*P* < 0.05). Then, we included the above eight independent variables in a multivariate collinearity model to investigate the relationship between stalk mechanical properties and anatomical phenotypes, yielding the following full model: lm (formula = BS ~ PA + CAR-3 + TA + PAL + AAL + CCR + SA + AR). After stepwise multiple regression analysis of the full model, the optimal model contained two organ-level phenotypic variables (AAD and CCR) and three cell-level phenotypic variables (PA, CAR-3, and TA). The statistically significant variables were CAR-3, PA, and AAD. This model accounted for 77.7% of the variation. The influence of organ-level phenotypic variables on mechanical properties was very significant, which might mask the contribution of cell-level phenotypic variables.

In order to verify these results, we conducted modeling and an analysis of combinations of variables. First, the most significant macro variable (AAD) and other linear organ-level variables associated with mechanical properties (PAL, CCR, SA, and AR) were selected to establish a model for regression analysis (formula = BS ~ AAD + PAL + CCR + SA + AR). After stepwise multiple regression analysis of the full model, the optimal model was found to contain PAD, AR, and CCR variables, and this model accounted for 52.16% of the variation. Second, the significant micro variables CAR-3, PA, and TA were selected to establish a second model for regression analysis (formula = BS ~ CAR-3 + PA + TA). After stepwise multiple regression analysis of the full model, the optimal model contained CAR-3 and PA variables, and this model accounted for 68.93% of the variation. Third, macro variables and micro variables were selected to establish the combination model for further regression analysis (formula = BS ~ CAR-3 + PA + TA + AAD). The results of the stepwise regression analysis showed that the model was the optimal model, and the variables in the model were significantly correlated with mechanical properties (*P* < 0.05), which accounted for 77.09% of the variation (Table 2). In summary, the most relevant variables corresponding to mechanical properties were vascular bundle total area in the peripheral layer (PA), auxiliary axis diameter of the stalk cross-section (AAD), vascular bundle area ratio of the central third layer (CAR-3), and total vascular bundle area (TA). Moreover, our results suggest that anatomical phenotypes including cell-level and organ-level phenotypic characteristics were much better predictors of stalk mechanical properties than the use of organ-level morphological indexes alone.

**Table 2:** Summary of multiple regression model for stalk mechanical properties as predicted by macro variables and micro variables

	Estimate	Std. error	*t*-value	Pr (> |t|)	
(Intercept)	− 206.117	114.139	− 1.806	0.080358*	
PA	20.954	4.605	4.55	7.30E−05***	
CAR-3	− 3915.9	808.118	− 4.846	3.11E−05***	
TA	− 8.348	3.899	− 2.141	0.039993*	
AAD	23.404	6.242	3.75	0.000704***	
R^2^	0.7964				
Adjusted R^2^	0. 7709				

* *P* < 0.05

** *P* < 0.01

*** *P* < 0.001

## Discussion

Quantitative measures of plant morphology and anatomy are critical for understanding function [41]. Traditional micro-phenotypic traits analyses were of limited use for obtaining necessary details across large samples, posing severe barriers to the prediction of function–structure relationships [42]. To enable large-scale measurements of stalk anatomy features, image processing software and feature detection methods have been introduced, such as those by Legland et al. and Heckwolf et al. [20, 21]. Those tools have improved the efficiency of measuring vascular bundles significantly, but the lack of anatomical traits corresponding to the rind and resolution limitations have restricted accurate phenotyping. In 2016, VesselParser 1.0 was introduced, which made it possible to automatically analyze phenotypic traits of vascular bundles within entire cross-sections of maize stalks. This was the first time quantitative analysis of vascular bundle phenotypic traits within entire stalk cross-sections was feasible, and this image analysis tool can operate on CT images to measure anatomical features of vascular bundles in high throughput [22]. To obtain more phenotypic traits related to mechanics, version 2.0 has been improved and extended in our present work. VesselParser 2.0 displays several advantages, including performance accuracy, improved workflow organization, and efficiency by batch processing with prompted user interactions. Depending on the pixel size of the CT image, more accurate phenotypic features can be obtained. Certain measurements, such as measurements of peripheral vascular bundle total area (PA), central vascular bundle total area (CA), peripheral vascular bundle area ratio (PAR), central vascular bundle area ratio (CAR), vascular bundle average area (MA), and vascular bundle total area (TA), would be impractical to perform manually using Image J or Photoshop. Therefore, the traditional methods were limited to area measurements and any secondary variables that can be derived from area measurements. The performance of VesselParser 2.0 is improved, and it can extract more comprehensive phenotypic information from stalks, which presents an opportunity for further investigation of the value of these important anatomical traits.

Numerous studies have examined acute stress across changes in diameter [43, 44], and in this approach, calculations are based on the bending of an isotropic rod with an average cross-sectional parameter. Maize stalks are irregularly elliptical, and the average diameter influences the accuracy of the above calculation. In this research, principal axis diameter (PAD) and auxiliary axis diameter (AAD) were calculated using the image processing pipeline VesselParser 2.0, and the aspect ratio was also obtained. After the R statistical package data analysis, we found that the auxiliary axis diameter of the stalk was highly associated with the mechanical properties, which may be a better predictor of stalk mechanical properties than stalk average diameter. Correlation analysis provided further evidence that vascular bundle properties also played an important role in stalk mechanical properties [45]. Vascular bundles can act as a mechanical brace increasing the mechanical properties of stalks. The heterogeneous distributions of vascular bundles in cross-sectional internodes were displayed clearly in CT images, and the distribution trait in the rind region was significantly different from that in the center region. Vascular bundles of maize stalks were more densely distributed in the rind, thereby increasing the number of vascular bundles and their morphological size; thus, more fibrous tissue was available to distribute the bending forces. This information can yield new insights about functional–structure plant relationships and the role of the vascular bundle on stress distributions across maize stalks. Maize stalk pith has also been examined in research on stalk bending strength, and it generally prevents stalk cross-sections from ovalizing [46] and resists inward buckling of the rind tissue [43, 47]. Vascular bundles of much larger circumference are distributed in the cross-section of JD38 stalks, and this presence of the vascular bundle changes the structure of pith tissue, increasing fibrous tissue that might enhance the bending strength, too. The more intensive distribution of vascular bundles in JD38 stalks provides plants with a much sturdier structure against harsher wind and storm conditions.

Three-point bending experiments of plants stems have been used to investigate a wide variety of subjects, including types of failure [48], crop bending [43, 49], and correlations among cultivation measures, environmental impacts, and mechanical properties [31]. According to this research, the mechanical properties of any structure (including corn stalks) were determined by two governing factors, namely material properties and morphology (i.e., anatomical geometry) [49]. For most structures, shape (morphology) has a dominant effect on the bending response. In a collaboration integrating plant science and biomechanical engineering, Von Forell, et al. demonstrated that changes in stalk morphology had, on average, 18-fold more influence on stalk mechanical stresses than changes in material properties had [50]. Accordingly, we believe that understanding the relationships between anatomical phenotypes and bending properties of maize stalk will yield more valuable information on lodging-resistance. In the present experiment, after stepwise regression, the optimal model contained the organ-level phenotypic variable AAD and the cell-level phenotypic variable TA, indicating that these two variables had the highest correlation with mechanical properties. Auxiliary axis diameter (AAD) was found to be a better predictor of stalk mechanical properties than stalk average diameter. Moreover, auxiliary axis diameter (AAD) and vascular bundle total area (TA) were more precise predictors of stalk bending strength. The relationship between the anatomical phenotypes of total vascular bundles of stalks and the bending performance provide further evidence for fully understanding stalk biomechanical properties. It is necessary to consider the various phenotyping levels simultaneously and attempt to unravel causal relationships among them. Our results indicate that stalk micro-phenotypic characteristics should be considered and these parameters are important predictors of stalk biomechanical properties, which are more fundamental than aggregate traits like stalk diameter and thus may be improved selection criteria in crop breeding research.

## Conclusion

The development of high-throughput, highly automated phenotypic analysis software enables the measurement of dozens to hundreds of individuals within defined populations for the purpose of resistance selection or characterizing varieties. The high-throughput extraction of these phenotypic parameters is of great importance in exploring the relationship between stalk mechanical properties and anatomical structures. Our work demonstrates the successful application of VesselParser 2.0 in plant physiological research. There were significant differences in stalk microstructures between the two different varieties, especially in their vascular bundle anatomical characteristics, which were consistent with their contrasting lodging performance. The combination of anatomical and mechanical research provides unique insights into the problem of stalk lodging, and micro phenotypes provide important indices of maize stalk mechanical performance. Future extensions of Vessel Parser will focus on the automatic segmentation and extraction of more precise phenotypes such as those corresponding to conduits, phloem, and vascular sheath cells, which may provide even more fruitful selection criteria for enhancing stalk biomechanical properties.

## Abbreviations

ROI: regions of interest
JD38: Jingdan38
JK968: Jingke968
PCA: principal components analysis
AIC: akaike information criterion
PAD: principal axis diameter
AAD: auxiliary axis diameter
CCR: circumradius
SA: section area
VB: vascular bundle number
PVB: vascular bundle number of peripheral layer
CVB-2: vascular bundle number of central 2nd layer
CVB-3: vascular bundle number of central 3rd layer
CVB-4: vascular bundle number of central 4th layer
PA: vascular bundle total area in peripheral layer
CA-2: vascular bundle total area in central 2nd layer
CA-3: vascular bundle total area in central 3rd layer
CA-4: vascular bundle total area in central 4th layer
PAR: vascular bundle area ratio of peripheral layer
CAR-2: vascular bundle area ratio of central 2nd layer
CAR-3: vascular bundle area ratio of central 3rd layer
CAR-4: vascular bundle area ratio of central 4th layer
MA: vascular bundle average area
TA: vascular bundle total area
Rsection: ratio of vascular bundle total area and section area
AR: aspect ratio
Fmax: bending load at which the stalk breaks
